# Correction to: The Toronto cognitive assessment (TorCA): normative data and validation to detect amnestic mild cognitive impairment

**DOI:** 10.1186/s13195-018-0446-z

**Published:** 2018-12-07

**Authors:** Morris Freedman, Larry Leach, M. Carmela Tartaglia, Kathryn A. Stokes, Yael Goldberg, Robyn Spring, Nima Nourhaghighi, Tom Gee, Stephen C. Strother, Mohammad O. Alhaj, Michael Borrie, Sultan Darvesh, Alita Fernandez, Corinne E. Fischer, Jennifer Fogarty, Barry D. Greenberg, Michelle Gyenes, Nathan Herrmann, Ron Keren, Josh Kirstein, Sanjeev Kumar, Benjamin Lam, Suvendrini Lena, Mary Pat McAndrews, Gary Naglie, Robert Partridge, Tarek K. Rajji, William Reichmann, M. Uri Wolf, Nicolaas P. L. G. Verhoeff, Jordana L. Waserman, Sandra E. Black, David F. Tang-Wai

**Affiliations:** 1 Department of Medicine (Neurology), University of Toronto, Toronto, ON Canada; 2 Baycrest Health Sciences, 3560 Bathurst Street, Toronto, ON M6A 2E1 Canada; 3 Rotman Research Institute of Baycrest Centre, Toronto, ON Canada; 4 Toronto Dementia Research Alliance, Toronto, ON Canada; 5 Mt. Sinai Hospital, Toronto, ON Canada; 6 Department of Psychology, Glendon College, Toronto, ON Canada; 7 Toronto Western Hospital, University Health Network, Toronto, ON Canada; 8 Tanz Centre for Research in Neurodegenerative Diseases, Toronto, ON Canada; 9 Sunnybrook Research Institute, Toronto, ON Canada; 10 Department of Medical Biophysics, University of Toronto, Toronto, ON Canada; 11 Canada International Scientific Exchange Program, Toronto, ON Canada; 12 Lawson Health Research Institute, London, ON Canada; 13 Parkwood Institute, London, ON Canada; 14 Department of Medicine (Neurology and Geriatric Medicine) and Department of Medical Neuroscience, Dalhousie University, Halifax, NS Canada; 15 Keenan Research Centre for Biomedical Research, Li Ka Shing Knowledge Institute, St. Michael’s Hospital, Toronto, ON Canada; 16 University Health Network, Toronto, ON Canada; 17 Sunnybrook Health Sciences Centre, Toronto, ON Canada; 18 Department of Psychiatry, University of Toronto, Toronto, ON Canada; 19 Centre for Addiction and Mental Health, Toronto, ON Canada; 20 Hurvitz Brain Sciences Program, Sunnybrook Health Sciences Centre, Toronto, ON Canada; 21 Krembil Research Institute, University Health Network, Toronto, ON Canada; 22 Department of Psychology, University of Toronto, Toronto, ON Canada; 23 Department of Medicine (Geriatric Medicine) and Institute of Health Policy, University of Toronto, Toronto, ON Canada; 24 Campbell Family Mental Health Research Institute, Toronto, ON Canada; 25 LC Campbell Cognitive Neurology Research Unit, Toronto, ON Canada

## Erratum

Upon publication of this article [1], it was brought to our attention that one of the 303 participants in the normative study should have been deleted from the database. Therefore, we reanalyzed the data with this individual removed. This resulted in minor numerical changes affecting tables, figures, and text. In addition, we added IQ data that were omitted in seven participants with normal cognition. This resulted in minor changes affecting Table 9. There were also minor typographical corrections made in the tables.

There was no significant impact on the analyses or findings reported in the paper from any of the revisions. The changes are as follows:

Table 2

- Due to deletion of the single participant who should have been omitted from the database, the sample size was changed from 303 to 302 in the 50–89 year old group and from 76 to 75 in the 50–59 year old group. The number of males in each group was reduced by 1. The Mean (SD) TorCA Sum scores were revised in the 50–89 and 50–59 year old groups.
- The cut-off scores for the impaired, borderline, and normal limits ratings for the Sum Index were revised in the 50–59 year old group.
- The cut-off scores for the impaired and borderline ratings for the Delayed Memory Recognition Index were revised in the 70–79 year old group.
- The cut-off scores for the impaired and borderline ratings for the Visuospatial Index were revised in the 70–79 and 80–89 year old groups.

Table 4

- The cut-off scores for the below normal and borderline ratings for Clock Drawing were revised in the 50–89 year old group.

Table 5

- The cut-off score for the borderline rating for Digit Span Backwards was revised for the 70–79 year old group.
- The cut-off scores for the borderline and normal limits ratings for Digit Span Backwards were revised for the 80–89 year old group.

Table 6

- The cut-off score for the borderline rating for Repetition was revised for the 50–89 year old group.

Table 7

- The Test2-Test1 Mean Difference was revised from 2.8 to 2.4 for the Memory – Immediate Recall Index.

Table 9

- There was a revision to the demographic information in which IQ data for seven participants with normal cognition were omitted. With the addition of these seven participants, there was a change in the Mean IQ (SD). The t-test comparing the IQ of participants with aMCI to those with normal cognition was recalculated with these seven individuals included. There was a minor change in the degrees of freedom and the *p*-value.
- One participant with aMCI was not given the verbal component of the IQ estimate due to non-exclusionary English as a second language considerations. However, a comparable estimate of IQ was within the range exhibited by the remaining aMCI participants. This was added in a footnote.

Figure 1

- The sample size was changed from 303 to 302

Figure 4

Due to a change in cut-off scores:

- The rating for MDRec in the 70–79 year old group was changed from an orange triangle to a blue dot, i.e., from below normal limits to borderline.
- The rating for MDRec in the Index Plot was changed from an orange triangle to a blue dot, i.e., from below normal limits to borderline.

Text (page 5, column 2, paragraph 2)

Due to the change in sample size from 303 to 302, there was a change in the degrees of freedom, F values, Cohen’s d, and number of points higher on Sum Index in women than men. The revised text is:

The Sum Index was significantly affected by age (F(3,298) = 7.27, *p* = 0.001) (Table 2). There was a significant but small effect size (Cohen’s d = 0.29) [20] for gender. Women scored a mean of 5.5 (SED = 2.2) points higher than men (F(1,300) = 6.24, *p* = 0.013). Age and education were weakly, but significantly, correlated with Sum Index (*r* = 0.24 and 0.23, both *p* < 0.001), each accounting for approximately 5% of the variance.

The revised tables and figures are shown on the following pages.

The revised tables are:

**Table 2:** Toronto Cognitive Assessment (TorCA) group profiles and normative data

Group profile			Age group				
			50–89 years	50–59 years	60–69 years	70–79 years	80–89 years
*N*			302	75	77	75	75
Male/female			103/199	28/47	22/55	20/55	33/42
Years of education, median (range)			16 (8–20)	16 (12–20)	16 (11–20)	16 (9–20)	14 (8–20)
TorCA Sum Index, mean (standard deviation)			292.8 (18.4)	297.6 (18.6)	296.9 (16.7)	290.5 (16.6)	286.0 (19.4)
TorCA Sum Index, median			295	301	298	291	289
Normative Data	Percentile range	Rating	50–89 years	50–59 years	60–69 years	70–79 years	80–89 years
Sum Index	≤ 5	Impaired	< 261	< 266	< 272	< 262	< 257
	6–24	Borderline	261–281	266–287	272–287	262–280	257–272
	≥ 25	Normal limits	> 281	> 287	> 287	> 280	> 272
Orientation	≤ 5	Impaired	< 10	< 10	< 10	< 10	< 10
	6–24	Borderline	10	10	10	10	10
	≥ 25	Normal limits	> 10	> 10	> 10	> 10	> 10
Immediate Memory Recall	≤ 5	Impaired	< 15	< 17	< 16	< 15	< 14
	6–24	Borderline	15–18	17–20	16–18	15–17	14–16
	≥ 25	Normal limits	> 18	> 20	> 18	> 17	> 16
Delayed Memory Recall	≤ 5	Impaired	< 10	< 14	< 12	< 8	< 6
	6–24	Borderline	10–14	14–16	12–15	8–12	6–12
	≥ 25	Normal limits	> 14	> 16	> 15	> 12	> 12
Delayed Memory Recognition	≤ 5	Impaired	< 19	< 20	< 19	< 18	< 18
	6–24	Borderline	19	20	19	18–19	18
	≥ 25	Normal limits	> 19	21	> 19	> 19	> 18
Visuospatial	≤ 5	Impaired	< 25	< 27	< 25	< 24	< 24
	6–24	Borderline	25–27	27–28	25–27	24–27	24–27
	≥ 25	Normal limits	> 27	> 28	> 27	> 27	> 27
Working Memory/Attention/Executive Control	≤ 5	Impaired	< 99	< 98	< 102	< 99	< 98
	6–24	Borderline	99–106	98–105	102–107	99–106	98–105
	≥ 25	Normal limits	> 106	> 105	> 107	> 106	> 105
Language	≤ 5	Impaired	< 71	< 63	< 74	< 74	< 66
	6–24	Borderline	71–78	63–78	74–80	74–78	66–76
	≥ 25	Normal limits	> 78	> 78	> 80	> 78	> 76

**Table 4:** Normative data for subtests within domains: Visuospatial

		Toronto Cognitive Assessment Visuospatial test ratings	
Percentile	Rating	Benson Figure Copy	Clock Drawing
Ages 50–89 years			
≤ 5	Below normal	< 14	< 10
6–24	Borderline	14	10–12
≥ 25	Within normal limits	> 14	> 12
Ages 50–59 years			
≤ 5	Below normal	< 15	< 11
6–24	Borderline	15	11–12
≥ 25	Within normal limits	> 15	> 12
Ages 60–69 years			
≤ 5	Below normal	< 14	< 10
6–24	Borderline	14	10–12
≥ 25	Within normal limits	> 14	> 12
Ages 70–79 years			
≤ 5	Below normal	< 14	< 10
6–24	Borderline	14	10–12
≥ 25	Within normal limits	> 14	> 12
Ages 80–89 years			
≤ 5	Below normal	< 13	< 9
6–24	Borderline	13–14	9–12
≥ 25	Within normal limits	> 14	> 12

**Table 5:** Normative data for subtests within domains: Working Memory/Attention/Executive Control

		Toronto Cognitive Assessment Working Memory/Attention/Executive Control Test Ratings												
Percentile	Rating	Serial Subtractions 7 s	Serial Subtractions 3 s	Serial Subtractions Total	Digit Span Forwards	Digit Span Backwards	Digit Span Total	Trails A Time	Trails A Score	Trails B Time	Trails B Score	Trails Time Difference	Alternating Sequences	Similarities
Ages 50–89 years														
≤ 5	Below normal	< 9	< 11	< 21	< 5	< 4	< 10	> 67	< 24	> 163	< 22	> 107	< 2	< 7
6–24	Borderline	9–10	11–12	21–23	5	4	10	67–47	–	163–107	22	107–63	–	7–8
≥ 25	Within normal limits	> 10	> 12	> 23	> 5	> 4	> 10	< 47	24	< 107	> 22	< 63	2	> 8
Ages 50–59 years														
≤ 5	Below normal	< 9	< 11	< 21	< 5	< 4	< 10	> 67	< 24	> 163	< 22	> 107	< 2	< 7
6–24	Borderline	9–10	11–12	21–23	5	4	10	67–47	–	163–107	22	107–63	–	7–8
≥ 25	Within normal limits	> 10	> 12	> 23	> 5	> 4	> 10	< 47	24	< 107	> 22	< 63	2	> 8
Ages 60–69 years														
≤ 5	Below normal	< 10	< 11	< 21	< 5	< 4	< 9	> 59	< 24	> 146	< 24	> 100	< 2	< 9
6–24	Borderline	10	11–12	21–23	5	4	9–10	59–43	–	146–91	–	100–53	–	9
≥ 25	Within normal limits	> 10	> 12	> 23	> 5	> 4	> 10	< 43	24	< 91	24	< 53	2	> 9
Ages 70–79 years														
≤ 5	Below normal	< 9	< 11	< 20	< 5	< 4	< 10	> 86	< 24	> 196	< 23	> 137	0	< 8
6–24	Borderline	9–11	11	20–23	5	4	10	86–49	–	196–111	23	137–65	1	8
≥ 25	Within normal limits	> 11	> 11	> 23	> 5	> 4	> 10	< 49	24	< 111	24	< 65	2	> 8
Ages 80–89 years														
≤ 5	Below normal	< 9	< 11	< 22	< 5	< 4	< 9	> 73	< 24	> 198	< 21	> 159	0	< 7
6–24	Borderline	9–10	11–12	22–23	5	4	9	73–53	–	198–120	21–22	159–85	1	7–8
≥ 25	Within normal limits	> 10	> 12	> 23	> 5	> 4	> 9	< 53	24	< 120	> 22	< 85	2	> 8

**Table 6:** Normative data for subtests within domains: Language

		Toronto Cognitive Assessment Language Test Ratings:								
Percentile	Rating	F-words	Animal names	Naming	Repetition	Single word comprehension	Reading single word comprehension	Sentence comprehension	Single word reading	Semantic knowledge
Ages 50–89 years										
≤ 5	Below normal limits	< 10	< 14	< 13	< 8	< 8	< 2	< 5	< 11	< 9
6–24	Borderline	10–12	14–16	13	8	–	–	5–6	11	9
≥ 25	Normal limits	> 12	> 16	> 13	> 8	8	2	> 6	12	> 9
Ages 50–59 years										
≤ 5	Below normal limits	< 8	< 13	< 9	< 5	< 8	< 2	< 5	< 9	< 9
6–24	Borderline	8–11	13–18	9–13	5–7	–	–	5–6	9–11	9
≥ 25	Normal limits	> 11	> 18	> 13	> 7	8	2	> 6	12	10
Ages 60–69 years										
≤ 5	Below normal limits	< 10	< 14	< 13	< 8	< 8	< 2	< 6	< 12	< 9
6–24	Borderline	10–12	14–17	13	8	–	–	6–7	–	9
≥ 25	Normal limits	> 12	> 17	> 13	> 8	8	2	8	12	10
Ages 70–79 years										
≤ 5	Below normal limits	< 10	< 14	< 13	< 8	< 8	< 2	< 5	< 12	< 9
6–24	Borderline	10–12	14–16	13	8	–	–	5–6	–	9
≥ 25	Normal limits	> 12	> 16	> 13	> 8	8	2	> 6	12	10
Ages 80–89 years										
≤ 5	Below normal limits	< 11	< 11	< 12	< 8	< 8	< 2	< 4	< 11	< 9
6–24	Borderline	11–12	11–15	12	8	–	–	4–5	11	9
≥ 25	Normal limits	> 12	> 15	> 12	> 8	8	2	> 5	12	10

**Table 7:** Toronto Cognitive Assessment (TorCA) Test–Retest Results

TorCA index	Test 1 mean (SE)	Test 2 mean (SE)	Test 2–Test 1 mean difference (SED)	*t*(27) (*p* value)	Stability (*p* value)	% change
Orientation	11.2 ± 0.2	11.3 ± 0.2	0.1 ± 0.2	0.5 (0.631)	0.10 (0.607)	0.1
Memory—Immediate Recall	19.5 ± 0.7	21.9 ± 0.7	2.4 ± 0.5	4.6 (0.0001)	0.73 (0.0001)	14.3
Memory—Delayed Recall	15.8 ± 0.9	17.5 ± 0.8	1.7 ± 0.5	3.4 (0.002)	0.83 (0.0001)	10.7
Memory—Delayed Recognition	20.2 ± 0.2	20.4 ± 0.2	0.2 ± 0.2	0.9 (0.363)	0.57 (0.001)	1.0
Visuospatial	28.6 ± 0.4	28.4 ± 0.4	− 0.2 ± 0.3	− 0.7 (0.5)	0.68 (0.0001)	0.7
Executive Control^a^	111.0 ± 1.2	112.0 ± 1.3	1.0 ± 1.3	0.9 (0.4)	0.52 (0.004)	1.0
Language	84.4 ± 1.3	83.1 ± 1.3	− 1.3 ± 0.9	− 1.4 (0.2)	0.75 (0.0001)	1.5
Sum	290.7 ± 3.2	294.0 ± 3.4	3.3 ± 1.4	2.4 (0.023)	0.92 (0.0001)	1.1

Test 1 and Test 2 mean indices and test–retest correlations (test stability) expressed as Pearson *r*

Interpretation of stability coefficients (Pearson r): very good, ≥ 0.90; good, 0.80–0.89; acceptable, 0.70–0.79; low, < 0.70

*SE* standard error, *SED* standard error of the difference

^a^Working Memory/Attention/Executive Control

**Table 9:** Normal cognition and aMCI group demographics and TorCA indices comparisons

Group demographics	NC	aMCI		
*N*	57	50		
Male/female	19/38	27/23	*χ*^*2*^ = 4.6 *p* = 0.031	
Age, mean (SD)	75.3 (7.9)	77.7 (6.5)	*t*(105) *=* 1.68 *p* = 0.097	
Years of education, mean (SD)	15.02 (3.2)	15.5 (3.4)	*t*(105) = 0.72 *p* = 0.47	
IQ, mean (SD)	122.81 (13.54)*	121.33 (13.98)	*t*(104) = 0.55 *p* = 0.58	
TorCA index group comparisons	NC (SD)	aMCI (SD)	*t*(105) (*p* value**)	Effect size, Hedge’s *g* (95% CI)
Orientation	11.58 (0.76)	10.38 (1.69)	4.84 (0.0001)	− 0.93 (− 1.33, − 0.53)
Memory—Immediate Recall	20.77 (4.45)	14.18 (3.29)	8.62 (0.0001)	− 1.66 (− 2.10, − 1.22)
Memory—Delayed Recall	16.86 (4.85)	6.66 (4.65)	11.07 (0.0001)	− 2.13 (− 2.60, − 1.65)
Memory—Delayed Recognition	20.19 (1.33)	17.42 (2.42)	7.45 (0.0001)	−1.43 (− 1.86, − 1.01)
Visuospatial	29.79 (1.80)	30.02 (2.16)	0.602 (0.549)	0.12 (− 0.26, 0.50)
Working Memory/Attention/Executive Control	108.47 (10.30)	107.34 (8.17)	0.625 (0.534)	− 0.12 (− 0.50, 0.26)
Language	80.16 (8.34)	76.90 (6.23)	2.26 (0.026)	− 0.42 (− 0.81, − 0.04)
Sum	287.82 (23.92)	262.86 (17.63)	6.07 (0.0001)	− 1.17 (− 1.58, − 0.76)

*aMCI* amnestic mild cognitive impairment, *CI* confidence interval, *NC* normal cognition, *SD* standard deviation, *TorCA* Toronto Cognitive Assessment

*One participant with aMCI was not given the verbal component of the IQ estimate due to non-exclusionary English as a second language considerations. A comparable estimate of IQ was within the range exhibited by the remaining aMCI participants

**Significance tests corrected for multiple comparisons using Bonferroni correction at *p* ≤ 0.05/7 (0.007)

The revised figures are:

**Fig. 1:**
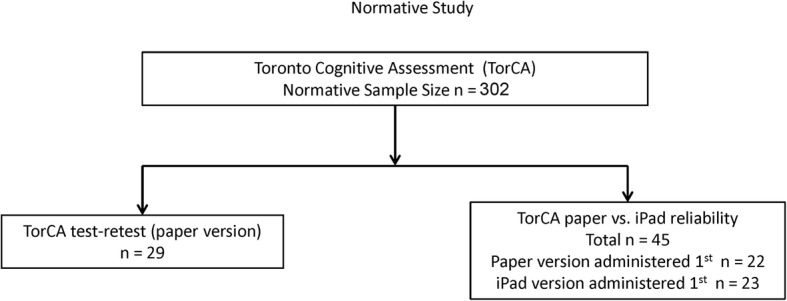
Flow chart of participants for normative study

**Fig. 4:**
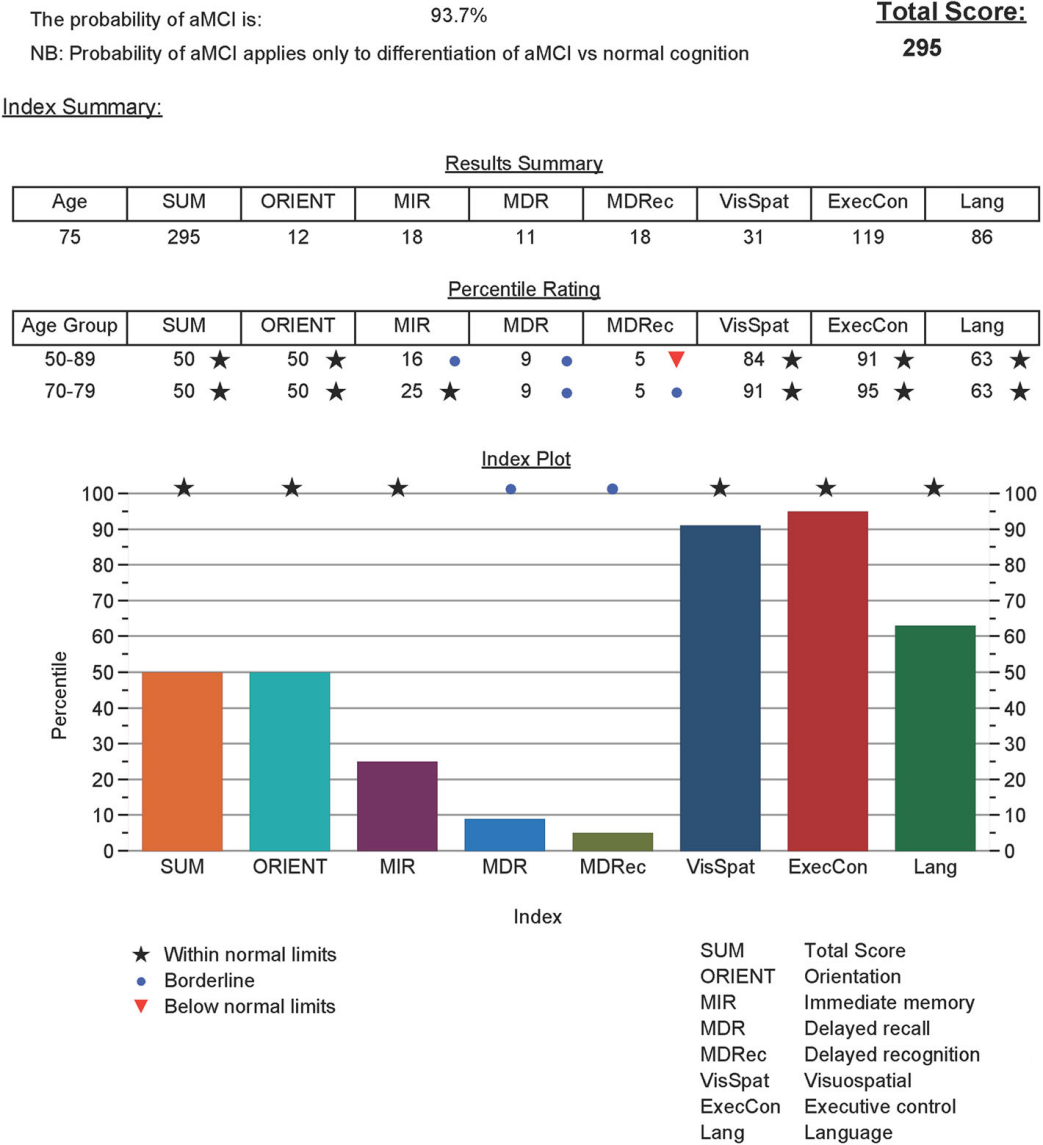
iPad summary score sheet showing domain scores and numerical and graphic percentile ratings. Probability of aMCI shown as 93.7%. aMCI amnestic mild cognitive impairment

In addition to the above, we have provided an annotated pdf as a Additional file 1 documenting the changes. The original article can be found online at 10.1186/s13195-018-0382-y

## Additional file

Additional file 1: Annotated pdf documenting changes to original article. (PDF 1130 kb)

## References

[1] Freedman M (2018). The Toronto Cognitive Assessment (TorCA): normative data and validation to detect amnestic mild cognitive impairment. Alzheimers Res Ther.

