# Supplementary material for: Correction to: The Toronto cognitive assessment (TorCA): normative data and validation to detect amnestic mild cognitive impairment
Source: Alzheimers Res Ther. 2018 Dec 7;10:120. doi: 10.1186/s13195-018-0446-z (PMC6286597; doi:10.1186/s13195-018-0446-z)
Supplement: Supplementary file 1 — Annotated pdf documenting changes to original article. (PDF 1130 kb) [file 13195_2018_446_MOESM1_ESM.pdf]

Freedman et al. Alzheimer's Research & Therapy (2018) 10:65
https://doi.org/10.1186/s13195-018-0382-y

Alzheimer's Research & Therapy

RESEARCH Open Access

The Toronto Cognitive Assessment (TorCA): normative data and validation to detect amnestic mild cognitive impairment

Morris Freedman[1,2,3,4,5*†], Larry Leach[4,6†], M. Carmela Tartaglia[1,4,7,8], Kathryn A. Stokes[2], Yael Goldberg[2], Robyn Spring[3], Nima Nourhaghighi[4,9], Tom Gee[3], Stephen C. Strother[3,4,10], Mohammad O. Alhaj[2,11], Michael Borrie[12,13], Sultan Darvesh[14], Alita Fernandez[2], Corinne E. Fischer[4,15,18], Jennifer Fogarty[12,13], Barry D. Greenberg[4,16], Michelle Gyenes[2], Nathan Herrmann[4,9,17,18], Ron Keren[4,16,18], Josh Kirstein[2], Sanjeev Kumar[4,18,19], Benjamin Lam[1,17,20], Suvendrini Lena[1,4,19], Mary Pat McAndrews[7,21,22], Gary Naglie[2,3,4,23], Robert Partridge[7], Tarek K. Rajji[4,18,19,24], William Reichmann[2,4,18], M. Uri Wolf[2,4,18], Nicolaas P. L. G. Verhoeff[2,4,18], Jordana L. Waserman[2], Sandra E. Black[1,4,9,17,20,25†] and David F. Tang-Wai[1,4,7,19,21†]

Abstract

Background: A need exists for easily administered assessment tools to detect mild cognitive changes that are more comprehensive than screening tests but shorter than a neuropsychological battery and that can be administered by physicians, as well as any health care professional or trained assistant in any medical setting. The Toronto Cognitive Assessment (TorCA) was developed to achieve these goals.

Methods: We obtained normative data on the TorCA ($n = 303$), determined test reliability, developed an iPad version, and validated the TorCA against neuropsychological assessment for detecting amnestic mild cognitive impairment (aMCI) ($n = 50/57$, aMCI/normal cognition). For the normative study, healthy volunteers were recruited from the Rotman Research Institute registry. For the validation study, the sample was comprised of participants with aMCI or normal cognition based on neuropsychological assessment. Cognitively normal participants were recruited from both healthy volunteers in the normative study sample and the community.

Results: The TorCA provides a stable assessment of multiple cognitive domains. The total score correctly classified 79% of participants (sensitivity 80%; specificity 79%). In an exploratory logistic regression analysis, indices of Immediate Verbal Recall, Delayed Verbal and Visual Recall, Visuospatial Function, and Working Memory/Attention/Executive Control, a subset of the domains assessed by the TorCA, correctly classified 92% of participants (sensitivity 92%; specificity 91%). Paper and iPad version scores were equivalent.

Conclusions: The TorCA can improve resource utilization by identifying patients with aMCI who may not require more resource-intensive neuropsychological assessment. Future studies will focus on cross-validating the TorCA for aMCI, and validation for disorders other than aMCI.

Keywords: Toronto Cognitive Assessment, TorCA, Mild cognitive impairment, Cognitive assessment, Diagnosis, Validation, Normative study

* Correspondence: mfreedman@baycrest.org
†Morris Freedman, Larry Leach, Sandra E. Black and David F. Tang-Wai contributed equally to this work.
[1]Department of Medicine (Neurology), University of Toronto, Toronto, ON, Canada
[2]Baycrest Health Sciences, 3560 Bathurst Street, Toronto, ON M6A 2E1, Canada
Full list of author information is available at the end of the article

© The Author(s). 2018 **Open Access** This article is distributed under the terms of the Creative Commons Attribution 4.0 International License (http://creativecommons.org/licenses/by/4.0/), which permits unrestricted use, distribution, and reproduction in any medium, provided you give appropriate credit to the original author(s) and the source, provide a link to the Creative Commons license, and indicate if changes were made. The Creative Commons Public Domain Dedication waiver (http://creativecommons.org/publicdomain/zero/1.0/) applies to the data made available in this article, unless otherwise stated.

Background

Brief tests such as the Mini-Mental State Examination (MMSE) [1] and the Montreal Cognitive Assessment (MoCA) [2] are popular screens for cognitive function. Neuropsychological assessments facilitate better understanding of cognitive performance for diagnosis but are time consuming, resource intensive, and suited for administration only by neuropsychologists—a resource that is often not readily available. Consequently, given the growing emphasis on early detection of cognitive impairment, there is a need for assessment tools that are intermediate between brief screening tests and neuropsychological batteries, can be administered by physicians as well as any health care professional or trained assistant in any medical setting, and can accurately identify mild cognitive decline. To accomplish this goal, the psychometric properties of the Behavioural Neurology Assessment [3], a screening test covering a broad spectrum of cognitive functions for diagnosing mild to moderate dementia, were significantly enhanced to detect mild cognitive deficits by development of the Toronto Cognitive Assessment (TorCA). This was done through the addition of more robust verbal learning and delayed recall, a complex figure copy with delayed recall, semantic knowledge items, a version of Trails A and B, and revision of the subset of language tests.

Our objectives were to obtain normative data on the TorCA and to validate this test for detection of amnestic mild cognitive impairment (aMCI). In addition to the paper version, we developed an electronic application for the iPad and assessed equivalency between the two versions. The advantages of an electronic application include automatic scoring, automatic point-of-care data collection for potential data entry into a clinical or research registry, a printable summary of results, and graphical representation of percentile performance on each cognitive domain.

Methods

Test description

The TorCA consists of 27 subtests within seven cognitive domains—Orientation, Immediate Recall, Delayed Recall, Delayed Recognition, Visuospatial Function, Working Memory/Attention/Executive Control, and Language (Table 1)—and can be administered by any health care professional or trained assistant and is suitable for use in any medical setting. Domain index scores represent addition of subtest scores within each domain. The Sum Index represents addition of all subtest scores.

1. Orientation

There are 12 items included: year, month, day, date, season, place/building, floor, city, province, country, Prime Minister, and Premier of the province.

2. Immediate Verbal Recall

The CERAD 10-Word list [4] is presented over three trials.

3. Delayed Verbal and Visual Recall

Delayed recall of the CERAD Word List and the Benson Figure Copy [5] are assessed after at least 10 min.

4. Delayed Verbal and Visual Recognition

Recognition of whether words appeared in the CERAD list and which one of four complex figures was copied are assessed.

5. Visuospatial Function

This scale consists of Clock Drawing [6] and the Benson Figure Copy [5].

6. Working Memory/Attention/Executive Control

Working memory and attention are assessed by Digit Span and Serial Subtractions. Executive control [7] is assessed by drawing Alternating Sequences, Verbal Letter Fluency, and Trail Making A and B [8]. A left–right reversed version of Trail Making is used to reduce practice effects on the standard version.

7. Language

There are eight subtests included: Verbal Fluency (animal names), confrontation naming of 15 items from the Multilingual Naming Test (MINT) [9], Sentence Repetition, Sentence Comprehension, Single Word Reading and Comprehension (auditory and reading), and Semantic Knowledge.

8. TorCA Sum Index

Consistent with standard practice in neuropsychology, there is no upper limit on Verbal Fluency for "F" words and animals. Therefore, there is no maximum on the Sum Index.

Standardization and normative sample

The study was approved by the Research Ethics Board at Baycrest Health Sciences. Healthy volunteers ($n = 303$) were recruited from the Rotman Research Institute (RRI) registry. There were four age groups: 50–59, 60–69, 70–79, and 80–89 years. Exclusion criteria were history of neurological disease, drug abuse, head injury with loss of consciousness, attention deficit hyperactivity

Table 1 Cognitive domains and scores on the Toronto Cognitive Assessment

Domain	Subtest	Subtest score	Maximum score for domain
Orientation			
	Orientation	12	12
Immediate Memory			
	CERAD Word List Trial 1	10	
	CERAD Word List Trial 2	10	
	CERAD Word List Trial 3	10	30
Delayed Recall			
	CERAD—Delayed Recall	10	
	Benson Figure Delayed Recall	17	27
Delayed Recognition			
	CERAD Delayed Recognition	20	
	Benson Figure Delayed Recognition	1	21
Visuospatial			
	Benson Figure Copy	17	
	Clock Drawing	15	32
Working Memory/Attention/Executive Control			
	Serial 7 s	13	
	Serial 3 s	13	
	Digit Span—Forward	9	
	Digit Span—Reverse	8	
	Trails A	24	
	Trails B	24	
	Alternating Sequences	2	
	Similarities	10	
	Verbal Fluency—F words	N/A	N/A
Language			
	Verbal Fluency—Animals	N/A	
	MINT Naming	15	
	Repetition	10	
	Single Word Comprehension	8	
	Single Word Reading Comprehension	2	
	Sentence Comprehension	8	
	Single Word Reading	12	
	Semantic Knowledge	10	N/A

CERAD Consortium to Establish a Registry for Alzheimer's Disease, *MINT* Multilingual Naming Test, *N/A* not applicable

disorder, active psychiatric illness, or use of medication containing any opioid. Non-native English speakers were included if they could understand all instructions. For test items, and administration and scoring instructions, see the Toronto Dementia Research Alliance website (www.tdra.ca). Figure 1 shows a flow chart of the participants analyzed in the normative study.

Reliability

To assess test stability, the TorCA was readministered to 29 participants after a median interval of 73 days (range 28–120) with mean difference, percentage score change, and stability coefficients (Pearson *r*) calculated between the first and second tests. Internal consistency was determined by calculating Cronbach's α for domain and Sum Index scores from the normative data study.

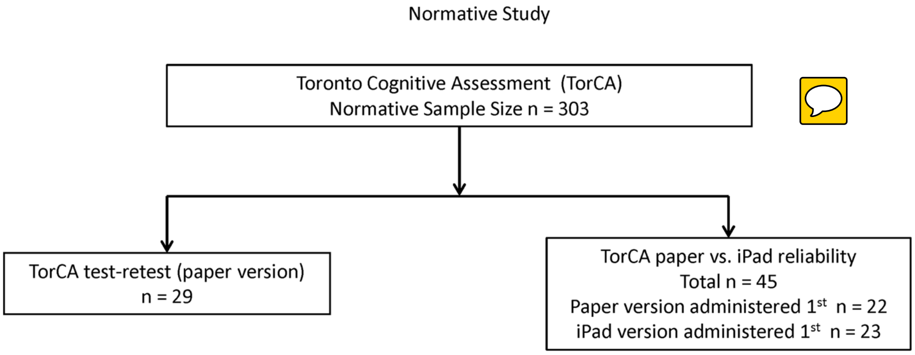

Fig. 1 Flow chart of participants for normative study

Validation in aMCI

Participants over age 60 years, with differential diagnosis of normal cognition vs MCI, were referred from academic memory clinics across Toronto and London, Ontario, for clinical neuropsychological assessment. Although differential diagnosis at referral may not have added the descriptor "amnestic" to MCI, the final study sample was comprised only of participants with aMCI or normal cognition (NC) based on neuropsychological assessment. From 220 consecutive referrals from all sites, 25 refused clinical services, 7 were inappropriate, and 188 were assessed by a neuropsychologist. Of those assessed, 108 did not have MCI or NC and four met exclusion criteria, yielding 50 participants with aMCI (single domain/multiple domain = 13/37) and 26 with NC. Figure 2 shows a flow chart of the participants analyzed in the validation study.

As it proved difficult to find individuals with normal cognition in memory clinics, the remaining 31 normal participants were recruited from the current normative study sample and the community. The paper version of the TorCA was administered prior to neuropsychological assessment in all but three instances. The interval between neuropsychological assessment and TorCA was within six months.

As assessments were conducted in a clinical context, the neuropsychologists were aware of the TorCA scores and differential diagnoses. The majority of neuropsychological assessments were conducted by trained assistants not directly involved in the diagnostic process, although one of the neuropsychologists tested 42 participants. The TorCA was conducted by trained nurses, medical trainees, or research assistants who were blinded to the neuropsychological assessment results.

Exclusion criteria for the validation study were medical or neurological disorders that could cause cognitive deficits including untreated sleep apnea, traumatic brain injury with loss of consciousness greater than 30 min, history of stroke, attention deficit hyperactivity disorder requiring medication, substance abuse, or other significant psychiatric disorders.

The following were administered as part of the neuropsychological battery:

- Kaplan–Baycrest Neurocognitive Assessment (KBNA) [10].
- Trail Making Test Forms A and B [8].
- Wechsler Adult Intelligence Scale—III (WAIS-III) Digit Symbol [11].
- WAIS—III Digit Span [11].
- Wechsler Memory Scale—Revised (WMS-R) Logical Memory I and II subtests (Story A or B) [12].
- Wechsler Abbreviated Scale of Intelligence (WASI) Vocabulary (split half), Similarities, and Matrix Reasoning subtests [13].
- Boston Naming Test (split half) [14].
- Delis–Kaplan Executive Function System (D-KEFS) Color-Word Interference Test [15].
- Multifactorial Metamemory Questionnaire—Memory Mistakes scale [16].
- Lawton and Brody ADL questionnaire [17].
- Hospital Anxiety and Depression Scale [18].

All participants with aMCI met published criteria [19]. Objective memory impairment was defined as deficits on three of four memory tests relative to expectations based on age, education, and intellectual status. Memory tests were WMS-R Logical Memory, KBNA Word List [10], KBNA Complex Figure, and WAIS-III Digit Symbol incidental recall [11]. Deficit was defined as 1.5 standard deviations below estimated IQ based on the two-subtest IQ estimate of the WASI. Memory deficits had to occur at encoding or retention stages. Isolated retrieval deficits were not sufficient for diagnosis of aMCI.

Concurrent validity was determined by the ability of the TorCA to discriminate between aMCI and NC participants. Construct validity was determined by correlations between TorCA subtests and neuropsychological tests in the aMCI and NC groups and by testing for expected group differences on TorCA indices and subtests.

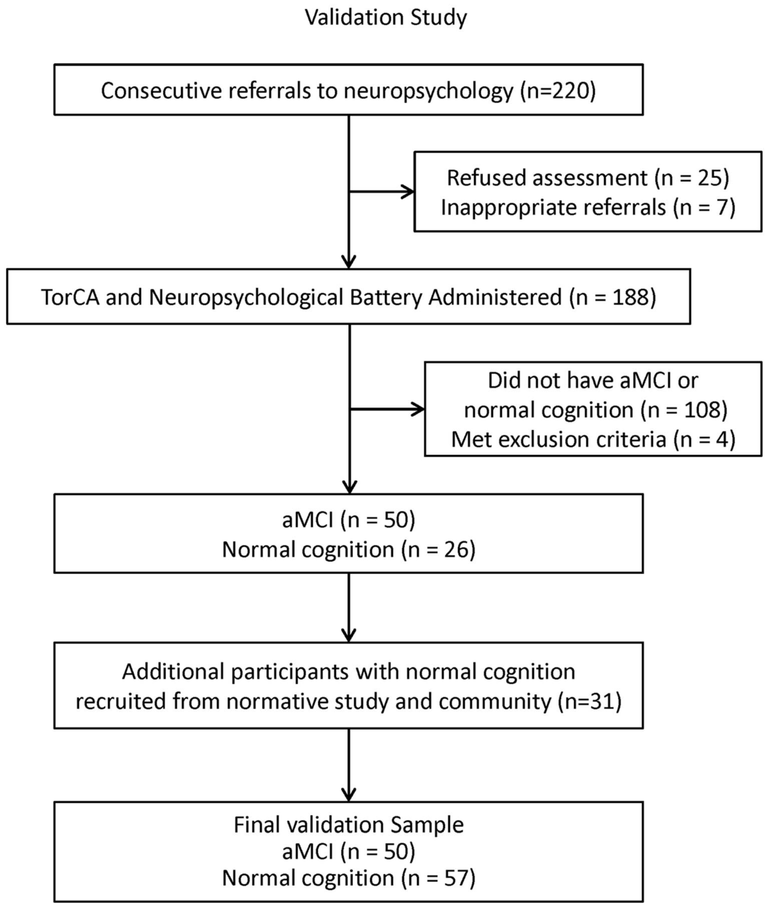

Fig. 2 Flow chart of participants for validation study. TorCA Toronto Cognitive Assessment, aMCI amnestic mild cognitive impairment

Equivalency of paper vs electronic version

Forty-five normal participants were tested using paper and iPad versions and were divided into two groups. One group ($n = 22$, female/male = 17/5; mean (SD) age = 73.6 (7.7) years) was recruited from the normative sample and was administered the paper version first (test–retest interval M = 792.3 days, SD = 262.9). The second group ($n = 23$, female/male = 18/5; mean (SD) age = 70.6 (10.1) years) was recruited from the RRI registry and was administered the iPad version first (test–retest interval M = 257.2 days, SD = 67.2).

Results

Normative study

Table 2 presents participant profiles and normative data. Groups did not differ in years of education. There were significantly more females for the 50–59 year group ($\chi^2(df = 1) = 4.26$, $p = 0.04$), 60–69 year group ($\chi^2(df = 1) = 14.14$, $p = 0.001$), and 70–79 year group ($\chi^2(df = 1) = 16.33$, $p = 0.001$) but not for the 80–89 year group ($\chi^2(df = 1) = 1.08$, $p = 0.30$).

Normative TorCA test scores are categorized into ≤ 5th percentile (impaired), 6th–24th percentile (borderline), or ≥ 25th percentile (normal). Median time to complete the TorCA was 34 min (range 25–63). Tables 3, 4, 5, and 6 present normative data for individual subtests.

The Sum Index was significantly affected by age (F(3,29~~9~~) = ~~6.45~~, $p = 0.001$) (Table 2). There was a significant but small effect size (Cohen's d = 0.~~31~~) [20] for gender. Women scored a mean of ~~6.1~~ (SED = 2.2) points higher than men (F(1,30~~1~~) = ~~7.36~~, p = 0.0~~07~~). Age and education were weakly, but significantly, correlated with Sum Index ($r = 0.24$ and 0.23, both $p < 0.001$), each accounting for approximately 5% of the variance.

The results of the test–retest study using the paper version in normal participants are presented in Table 7. The scores remained remarkably stable across the retest intervals. Only the Memory—Immediate Recall (MIR), Memory—Delayed Recall (MDR), and Sum Index scores demonstrated significant increases and the increase in the latter was due to increase in the MIR and MDR indices. This indicates that there was a practice effect on the memory tests. Stability coefficients ranged from low

Table 2 Toronto Cognitive Assessment (TorCA) group profiles and normative data

Group profile			Age group				
			50–89 years	50–59 years	60–69 years	70–79 years	80–89 years
N			303	76	77	75	75
Male/female			104/19[illegible]	29/47	22/55	20/55	33/42
Years of education, median (range)			16 (8–2[illegible]	16 (12–20)	16 (11–20)	16 (9–20)	14 (8–20)
TorCA Sum Index, mean (standard deviation)			292.6 (18.7)	296.8 (19.9)	296.9 (16.7)	290.5 (16.6)	286.0 (19.4)
TorCA Sum Index, median			295	301	298	291	289
Normative Data	Percentile range	Rating	50–89 years	50–59 years	60–69 years	70–79 years	80–89 years
Sum Index	≤ 5	Impaired	< 261	< 258	< 272	< 262	< 257
	6–24	Borderline	261–281	258–286	272–287	262–280	257–272
	≥ 25	Normal limits	> 281	> 286	> 287	> 280	> 272
Orientation	≤ 5	Impaired	< 10	< 10	< 10	< 10	< 10
	6–24	Borderline	10	10	10	10	10
	≥ 25	Normal limits	> 10	> 10	> 10	> 10	> 10
Immediate Memory Recall	≤ 5	Impaired	< 15	< 17	< 16	< 15	< 14
	6–24	Borderline	15–18	17–20	16–18	15–17	14–16
	≥ 25	Normal limits	> 18	> 20	> 18	> 17	> 16
Delayed Memory Recall	≤ 5	Impaired	< 10	< 14	< 12	< 8	< 6
	6–24	Borderline	10–14	14–16	12–15	8–12	6–12
	≥ 25	Normal limits	> 14	> 16	> 15	> 12	> 12
Delayed Memory Recognition	≤ 5	Impaired	< 19	< 20	< 19	< 19	< 18
	6–24	Borderline	19	20	19	19	18
	≥ 25	Normal limits	> 19	21	> 19	> 19	> 18
Visuospatial	≤ 5	Impaired	< 25	< 27	< 25	< 25	< 25
	6–24	Borderline	25–27	27–28	25–27	25–27	25–27
	≥ 25	Normal limits	> 27	> 28	> 27	> 27	> 27
Working Memory/Attention/Executive Control	≤ 5	Impaired	< 99	< 98	< 102	< 99	< 98
	6–24	Borderline	99–106	98–105	102–107	99–106	98–105
	≥ 25	Normal limits	> 106	> 105	> 107	> 106	> 105
Language	≤ 5	Impaired	< 71	< 63	< 74	< 74	< 66
	6–24	Borderline	71–78	63–78	74–80	74–78	66–76
	≥ 25	Normal limits	> 78	> 78	> 80	> 78	> 76

(Orientation and Memory—Delayed Recognition, Visuospatial, and Working Memory/Attention/Executive Control Indices) to very good (Sum Index). The poor stability coefficients of Orientation and Memory—Delayed Recognition, Visuospatial, and Working Memory/Attention/Executive Control in large part are due to a restricted range of scores.

The intratest reliabilities of the TorCA indices are presented in Table 8. Reliability estimates ranged from low to good. The low coefficients of Orientation, Memory—Delayed Recognition, and Visuospatial Indices again are attributable to the restricted range of scores noted earlier. The Delayed Recall Index reliability coefficient was calculated by comparing the results of the Memory—Delayed Verbal Recall and the Memory—Delayed Visual Recall subtests and therefore did not represent a homogeneous construct. The Visuospatial Index reliability coefficient was calculated by comparing the results of the Benson Figure Copy and Clock Drawing subtests. Although both Benson Figure Copy and Clock Drawing measure visuospatial function, Clock Drawing is also a measure of planning, monitoring, and abstraction. Thus, these subtests are not homogeneous. Likewise, the Working Memory/Attention/Executive Control Index is not homogeneous in construct as it consists of measures of attention, working memory, conceptualization, and reasoning.

Validation in aMCI

Table 9 presents demographic features of the aMCI and NC groups. The groups did not differ in mean

Table 3 Normative data for subtests within domains: Memory

		Toronto Cognitive Assessment Memory test ratings			
Percentile	Rating	Immediate recall	Verbal delayed recall	Verbal delayed recognition	Visual delayed recall
Ages 50–89 years					
≤ 5	Below normal	< 15	< 3	< 18	< 6
6–24	Borderline	15–18	3–4	18	6–8
≥ 25	Within normal limits	> 18	> 4	> 18	> 8
Ages 50–59 years					
≤ 5	Below normal	< 17	< 5	< 19	< 6
6–24	Borderline	17–20	5–6	19	6–9
≥ 25	Within normal limits	> 20	> 6	20	> 9
Ages 60–69 years					
≤ 5	Below normal	< 16	< 4	< 19	< 8
6–24	Borderline	16–18	4	19	8–9
≥ 25	Within normal limits	> 18	> 4	20	> 9
Ages 70–79 years					
≤ 5	Below normal	< 15	< 3	< 18	< 6
6–24	Borderline	15–17	3	18	6–7
≥ 25	Within normal limits	> 17	> 3	> 18	> 7
Ages 80–89 years					
≤ 5	Below normal	< 14	< 3	< 18	< 5
6–24	Borderline	14–16	3	18	5–7
≥ 25	Within normal limits	> 16	> 3	> 18	> 7

Table 4 Normative data for subtests within domains: Visuospatial

		Toronto Cognitive Assessment Visuospatial test ratings	
Percentile	Rating	Benson Figure Copy	Clock Drawing
Ages 50–89 years			
≤ 5	Below normal	< 14	~~< 11~~
6–24	Borderline	14	~~11–12~~
≥ 25	Within normal limits	> 14	> 12
Ages 50–59 years			
≤ 5	Below normal	< 15	< 11
6–24	Borderline	15	11–12
≥ 25	Within normal limits	> 15	> 12
Ages 60–69 years			
≤ 5	Below normal	< 14	< 10
6–24	Borderline	14	10–12
≥ 25	Within normal limits	> 14	> 12
Ages 70–79 years			
≤ 5	Below normal	< 14	< 10
6–24	Borderline	14	10–12
≥ 25	Within normal limits	> 14	> 12
Ages 80–89 years			
≤ 5	Below normal	< 13	< 9
6–24	Borderline	13–14	9–12
≥ 25	Within normal limits	> 14	> 12

Table 5 Normative data for subtests within domains: Working Memory/Attention/Executive Control

		Toronto Cognitive Assessment Working Memory/Attention/Executive Control test ratings												
Percentile	Rating	Serial Subtractions 7 s	Serial Subtractions 3 s	Serial Subtractions Total	Digit Span Forwards	Digit Span Backwards	Digit Span Total	Trails A Time	Trails A Score	Trails B Time	Trails B Score	Trails Time Difference	Alternating Sequences	Similarities
Ages 50–89 years														
≤ 5	Below normal	< 9	< 11	< 21	< 5	< 4	< 10	> 67	< 24	> 163	< 22	> 107	< 2	< 7
6–24	Borderline	9–10	11–12	21–23	5	4	10	67–47	–	163–107	22	107–63	–	7–8
≥ 25	Within normal limits	> 10	> 12	> 23	> 5	> 4	> 10	< 47	24	< 107	> 22	< 63	2	> 8
Ages 50–59 years														
≤ 5	Below normal	< 9	< 11	< 21	< 5	< 4	< 10	> 67	< 24	> 163	< 22	> 107	< 2	< 7
6–24	Borderline	9–10	11–12	21–23	5	4	10	67–47	–	163–107	22	107–63	–	7–8
≥ 25	Within normal limits	> 10	> 12	> 23	> 5	> 4	> 10	< 47	24	< 107	> 22	< 63	2	> 8
Ages 60–69 years														
≤ 5	Below normal	< 10	< 11	< 21	< 5	< 4	< 9	> 59	< 24	> 146	< 24	> 100	< 2	< 9
6–24	Borderline	10	11–12	21–23	5	4	9–10	59–43	–	146–91	–	100–53	–	9
≥ 25	Within normal limits	> 10	> 12	> 23	> 5	> 4	> 10	< 43	24	< 91	24	< 53	2	> 9
Ages 70–79 years														
≤ 5	Below normal	< 9	< 11	< 20	< 5	< 4	< 10	> 86	< 24	> 196	< 23	> 137	0	< 8
6–24	Borderline	9–11	11	20–23	5	–	10	86–49	–	196–111	23	137–65	1	8
≥ 25	Within normal limits	> 11	> 11	> 23	> 5	> 4	> 10	< 49	24	< 111	24	< 65	2	> 8
Ages 80–89 years														
≤ 5	Below normal	< 9	< 11	< 22	< 5	< 4	< 9	> 73	< 24	> 198	< 21	> 159	0	< 7
6–24	Borderline	9–10	11–12	22–23	5	–	9	73–53	–	198–120	21–22	159–85	1	7–8
≥ 25	Within normal limits	> 10	> 12	> 23	> 5	4	> 9	< 53	24	< 120	> 22	< 85	2	> 8

Table 6 Normative data for subtests within domains: Language

Percentile range	Rating	Toronto Cognitive Assessment Language Test Ratings: F-words	Animal names	Naming	Repetition	Single word comprehension	Reading single word comprehension	Sentence comprehension	Single word reading	Semantic knowledge
Ages 50–89 years										
≤ 5	Below normal limits	< 10	< 14	< 13	< 8	< 8	< 2	< 5	< 11	< 9
6–24	Borderline	10–12	14–16	13	–	–	–	5–6	11	9
≥ 25	Normal limits	> 12	> 16	> 13	> 8	8	2	> 6	12	> 9
Ages 50–59 years										
≤ 5	Below normal limits	< 8	< 13	< 9	< 5	< 8	< 2	< 5	< 9	< 9
6–24	Borderline	8–11	13–18	9–13	5–7	–	–	5–6	9–11	9
≥ 25	Normal limits	> 11	> 18	> 13	> 7	8	2	> 6	12	10
Ages 60–69 years										
≤ 5	Below normal limits	< 10	< 14	< 13	< 8	< 8	< 2	< 6	< 12	< 9
6–24	Borderline	10–12	14–17	13	8	–	–	6–7	–	9
≥ 25	Normal limits	> 12	> 17	> 13	> 8	8	2	8	12	10
Ages 70–79 years										
≤ 5	Below normal limits	< 10	< 14	< 13	< 8	< 8	< 2	< 5	< 12	< 9
6–24	Borderline	10–12	14–16	13	8	–	–	5–6	–	9
≥ 25	Normal limits	> 12	> 16	> 13	> 8	8	2	> 6	12	10
Ages 80–89 years										
≤ 5	Below normal limits	< 11	< 11	< 12	< 8	< 8	< 2	< 4	< 11	< 9
6–24	Borderline	11–12	11–15	12	8	–	–	4–5	11	9
≥ 25	Normal limits	> 12	> 15	> 12	> 8	8	2	> 5	12	10

age, education, or Full-Scale IQ. The NC group had a higher proportion of females (67%) to males (33%) (χ^2 = 6.33, $p < 0.02$), whereas the aMCI group had an approximately equal gender balance (54% male; 46% female).

Effect sizes based on difference between group means and standard deviations for neuropsychological tests used to determine group membership are provided in Fig. 3. There were significant effect sizes on verbal and visual learning (immediate recall of KBNA Word List and Complex Figure, WMS-R Logical Memory I), episodic memory (delayed recall and recognition of KBNA Word List and Complex Figure, WMS-R Logical Memory II), visual spatial working memory (KBNA Spatial Location), auditory working memory (WAIS-III Digit Span), attentional control (D-KEFS Color-Word Switching), visuospatial function (combined score for KBNA Complex Figure copy and Clock Drawing), semantic fluency (combined KBNA animal naming and first names), and cognitive flexibility (combined KBNA Practical Problem Solving and Conceptual Shifting). Overall, the aMCI group scored lower on neuropsychological testing but the largest effect sizes, in excess of 1.5 SD, were obtained on learning and episodic memory, thereby substantiating group classification as aMCI.

Table 9 presents between-group differences on TorCA indices. The aMCI group achieved a significantly lower TorCA Sum Index than did the NC group ($F(1,105) = 36.86$, $p < 0.001$). A MANOVA on the remaining seven domain indices revealed a significant effect for group (Wilk's $\lambda = 0.37$, $F(1,99) = 23.78$, $p < 0.001$). Pairwise comparisons, with Bonferroni correction for seven multiple comparisons at $p \leq 0.05/7$ (0.007), revealed significant differences for orientation, immediate memory recall, delayed memory recall, and delayed memory recognition indices.

Prior to analyzing TorCA subtest scores for group differences, boxplots for each subtest were inspected. Distribution of scores on Trail Making (completed trials measure, total correct minus incorrect lines), Alternating Sequences, Similarities, Sentence Repetition and

Table 7 Toronto Cognitive Assessment (TorCA) test–retest results

TorCA index	Test 1 mean (SE)	Test 2 mean (SE)	Test 2–Test 1 mean difference (SED)	*t*(27) (*p* value)	Stability (*p* value)	% change
Orientation	11.2 ± 0.2	11.3 ± 0.2	0.1 ± 0.2	0.5 (0.631)	0.10 (0.607)	0.1
Memory—Immediate Recall	19.5 ± 0.7	21.9 ± 0.7	2.8 ± 0.5	4.6 (0.0001)	0.73 (0.0001)	14.3
Memory—Delayed Recall	15.8 ± 0.9	17.5 ± 0.8	1.7 ± 0.5	3.4 (0.002)	0.83 (0.0001)	10.7
Memory—Delayed Recognition	20.2 ± 0.2	20.4 ± 0.2	0.2 ± 0.2	0.9 (0.363)	0.57 (0.001)	1.0
Visuospatial	28.6 ± 0.4	28.4 ± 0.4	− 0.2 ± 0.3	− 0.7 (0.5)	0.68 (0.0001)	0.7
Executive Control[a]	111.0 ± 1.2	112.0 ± 1.3	1.0 ± 1.3	0.9 (0.4)	0.52 (0.004)	1.0
Language	84.4 ± 1.3	83.1 ± 1.3	− 1.3 ± 0.9	− 1.4 (0.2)	0.75 (0.0001)	1.5
Sum	290.7 ± 3.2	294.0 ± 3.4	3.3 ± 1.4	2.4 (0.023)	0.92 (0.0001)	1.1

Test 1 and Test 2 mean indices and test–retest correlations (test stability) expressed as Pearson *r*
Interpretation of stability coefficients (Pearson r): very good, ≥ 0.90; good, 0.80–0.89; acceptable, 0.70–0.79; low, < 0.70
SE standard error, *SED* standard error of the difference
[a]Working Memory/Attention/Executive Control

Comprehension, Single Word Reading and Comprehension, and Semantic Knowledge showed a marked negative skew with a ceiling effect for both groups. Kolmogorov–Smirnov tests on these subtests revealed no differences in distribution of scores between the two groups. Therefore, these subtests were dropped from further between-group analyses.

Scores on Verbal Learning, Verbal Recall, Verbal Recognition, Visual Recall, Serial Subtractions, Digit Span, Trail Making A and B completed times measure, Benson Figure Copy, Clock Drawing, Verbal Fluency—F Words, Verbal Fluency—Animals, and MINT Naming were analyzed with a MANOVA for between-group differences (Table 10). There was a significant group effect (Wilk's $\lambda = 0.36$, $F(13,93)$, $p < 0.001$). Table 10 presents effect sizes for pairwise between-group comparisons for subtest scores. Large effect sizes, all in excess of 1.0, were obtained on memory tests including Verbal Learning, Delayed Verbal Recall, Delayed Verbal Recognition, and Delayed Visual Recall. There were moderate effect sizes on Trail Making B and Verbal Fluency—Animals. No significant between-group effects were found for Serial Subtractions, Trail Making A, Benson Figure Copy, Clock Drawing, Digit Span, Verbal Fluency—F Words, and MINT naming.

Concurrent validity with referenced neuropsychological tests

The TorCA Sum Index discriminated between the aMCI and NC groups ($\chi^2 = 31.5$, $p < 0.0001$, AUC = 0.84 (95% CI 0.75–0.92)). The sensitivity, specificity, likelihood ratio of a positive response (LRPR), likelihood ratio of a negative response (LRNR), positive predictive value (PPV), negative predictive value (NPV), Youden index, and correct classification of each Sum Index from 209 to 319 was calculated. The optimum cutoff value was determined by considering the maximum correct classification, LRPR, and Youden index combined with a view to minimizing false positives and maximizing classification accuracy. A Sum Index cutoff value of 275 was optimal and yielded an overall classification accuracy of 79% (95% CI 70–86%), sensitivity of 80% (95% CI 66–89%), specificity of 79% (95% CI 66–88%), LRPR of 3.80 (95% CI 2.26–6.40), and LRNR of 0.25 (95% CI 0.14–0.45). Given the aMCI prevalence of 47% in our sample, the 275 cutoff value yielded a PPV of 0.77 (95% CI 0.63–0.87) and NPV of 0.82 (95% CI 0.69–0.90). Agreement between the TorCA, using this cutoff value, and classification achieved by standard clinical and neuropsychological criteria was weak to moderate [21] ($\kappa = 0.58$ (95% CI 0.4–0.74)).

Table 8 Internal consistency of Toronto Cognitive Assessment (TorCA) indices

	TorCA index							
	Sum	Orientation	Immediate Memory	Delayed Recall	Delayed Recognition	Visuospatial	Executive Control[a]	Language
Consistency	0.73	0.20	0.81	0.62	0.38	0.60	0.51	0.74

Interpretation of internal consistency coefficients (Cronbach's α): very good, ≥ 0.90; good, 0.80–0.89; acceptable, 0.70–0.79; low, < 0.70
[a]Working Memory/Attention/Executive Control

Table 9 Normal cognition and aMCI group demographics and TorCA indices comparisons

Group demographics	NC	aMCI		
N	57	50		
Male/female	19/38	27/23	$\chi^2 = 4.6$<br>$p = 0.031$	
Age, mean (SD)	75.3 (7.9)	77.7 (6.5)	$t(105) = 1.68$<br>$p = 0.097$	
Years of education, mean (SD)	15.02 (3.2)	15.5 (3.4)	$t(105) = 0.72$<br>$p = 0.47$	
IQ, mean (SD)	122.3[illegible] (1[illegible].61)	121.33 (13.98)	$t(97) = 0.36$<br>$p = 0.72$	
TorCA index group comparisons	NC (SD)	aMCI (SD)	$t(105)$ (p value*)	Effect size, Hedge's g (95% CI)
Orientation	11.58 (0.76)	10.38 (1.69)	4.84<br>(0.0001)	− 0.93<br>(− 1.33, − 0.53)
Memory—Immediate Recall	20.77 (4.45)	14.18 (3.29)	8.62<br>(0.0001)	− 1.66<br>(− 2.10, − 1.22)
Memory—Delayed Recall	16.86 (4.85)	6.66 (4.65)	11.07<br>(0.0001)	− 2.13<br>(− 2.60, − 1.65)
Memory—Delayed Recognition	20.19 (1.33)	17.42 (2.42)	7.45<br>(0.0001)	−1.43<br>(− 1.86, − 1.01)
Visuospatial	29.79 (1.80)	30.02 (2.16)	0.602<br>(0.549)	0.12<br>(− 0.26, 0.50)
Working Memory/Attention/Executive Control	108.47 (10.30)	107.34 (8.17)	0.625<br>(0.534)	− 0.12<br>(− 0.50, 0.26)
Language	80.16 (8.34)	76.90 (6.23)	2.26<br>(0.026)	− 0.42<br>(− 0.81, − 0.04)
Sum	287.82 (23.92)	262.86 (17.63)	6.07<br>(0.0001)	− 1.17<br>(− 1.58, − 0.76)

aMCI amnestic mild cognitive impairment, *CI* confidence interval, *NC* normal cognition, *SD* standard deviation, *TorCA* Toronto Cognitive Assessment
*Significance tests corrected for multiple comparisons using Bonferroni correction at $p \leq 0.05/7$ (0.007)

To explore which TorCA indices best discriminated between aMCI and NC, indices for Orientation, Immediate Memory Recall, Delayed Memory Recall, Delayed Memory Recognition, Visuospatial, Working Memory/Attention/Executive Control, and Language were entered into a backward, stepwise logistic regression that generates a posttest probability of aMCI (Table 11). Four indices (Immediate Memory Recall, Delayed Memory Recall, Visuospatial, and Working Memory/Attention/Executive Control) correctly classified 92% (95% CI 86–97%) of the aMCI and NC groups (AUC = 97% (95% CI 94–99%)). Optimal discrimination was obtained for aMCI probability of 0.55, yielding sensitivity of 92% (95% CI 85–99%), specificity of 91% (95% CI 84–99%), PPV of 0.90 (95% CI 0.82–0.98), and NPV of 0.93 (95% CI 0.86–0.99). This corresponds to LRPR of 10.49 (95% CI 4.52–23.52) and LRNR of 0.09 (95% CI 0.03–0.23); both LRPR and LRNR values can yield large changes in posttest disease likelihood and thereby increase test accuracy [22, 23]. The indices in the logistic regression formula yielded strong agreement [21] with clinical and neuropsychological classification for aMCI ($\kappa = 0.83$ (95% CI 0.74–0.92), $\chi^2 = 74.0$, $p < 0.0001$).

Construct validity

The neuropsychological tests were grouped into nine domains: Immediate Recall, Delayed Recall, Delayed Recognition, Visuospatial, Cognitive Flexibility, Attention/Concentration, Executive Control, Verbal Fluency, and Language. Correlations between TorCA and neuropsychological domains are presented in Table 12. The largest correlations were obtained between the three TorCA memory domains and the three neuropsychological test domains relating to memory. Small to medium-sized effects were found between the TorCA memory domains and neuropsychological test domains of Cognitive Flexibility, Attention/Concentration, and Language. Large effect sizes were obtained between the TorCA Working Memory/Attention/Executive Control domain and the neuropsychological Working Memory/Attention/Executive Control, Verbal Fluency, and Language domains. Medium effect sizes were noted with the Cognitive Flexibility and Attention/Concentration

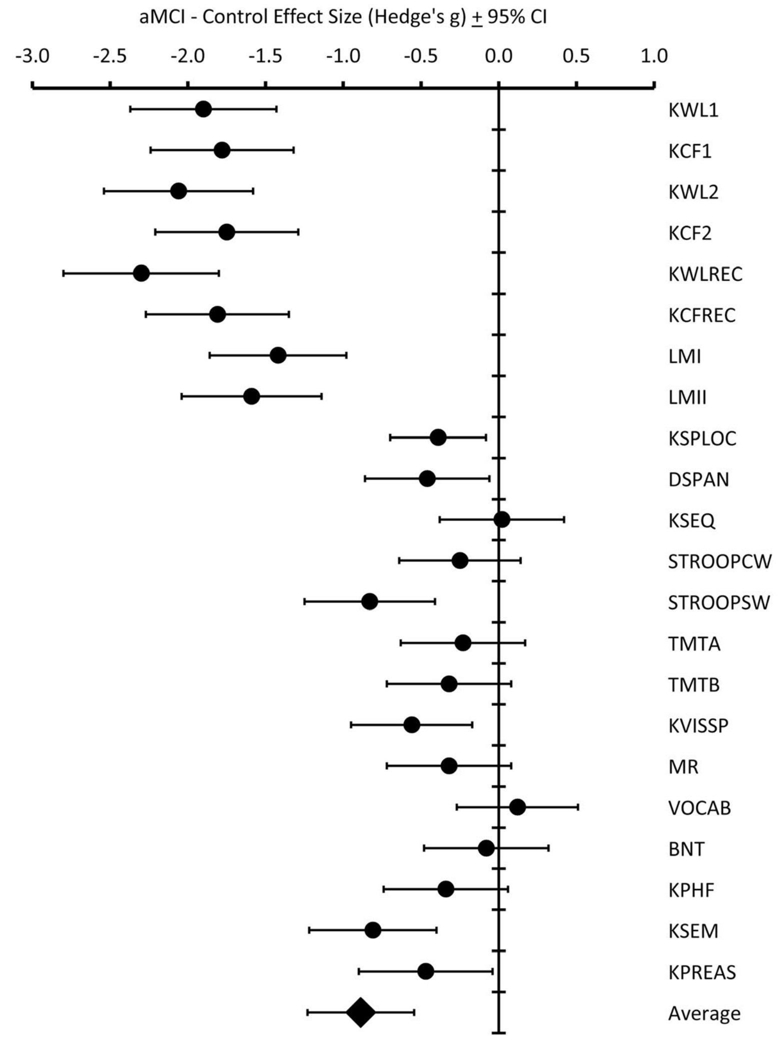

Fig. 3 Effect sizes on neuropsychological tests between aMCI and control groups. aMCI amnestic mild cognitive impairment, CI confidence interval, KWL1 KBNA Word List Learning immediate recall, KFC1 KBNA Complex Fig. 1 immediate recall, KWL2 KBNA Word List delayed recall, KFC2 KBNA Complex Figure delayed recall, KWLREC KBNA Word List delayed recognition, KCFREC KBNA Complex Figure delayed recognition, LMI WMS-III Logical Memory immediate recall, LMII WMS-III Logical Memory delayed recall, KSPLOC KBNA Spatial Location Memory, DSPAN WAIS-III Digit Span, KSEQ KBNA Sequencing, STROOPCW D-KEFS Color-Word Interference, STROOPSW D-KEFS Color-Word switching, TMTA Trail Making A, TMTB Trail Making B, KVISSP KBNA Complex Figure Copy + Clock Drawing, MR WAIS Matrix Reasoning, VOCAB WAIS Vocabulary, BNT Boston Naming Test, KPHF KBNA Phonemic Fluency, KSEM KBNA Semantic Fluency, KPREAS KBNA Practical Reasoning + Conceptual Shifting, KBNA Kaplan–Baycrest Neurocognitive Assessment, WMS-III Wechsler Memory Scale—IIII, WAIS-III Wechsler Adult Intelligence Scale—III, D-KEFS Delis–Kaplan Executive Function System

domains. The TorCA Working Memory/Attention/Executive Control domain was weakly associated with only the Immediate Recall domain. The Language domain was strongly associated with neuropsychological Language and Verbal Fluency domains, moderately associated with the Attention/Concentration, and Working Memory/Attention/Executive Control domains, and weakly associated with all three memory domains and Cognitive Flexibility. The TorCA Visuospatial domain showed a weak but significant correlation with the neuropsychological Visuospatial domain but no significant correlation with any other neuropsychological domain.

Equivalency of paper and iPad versions

There was a strong correlation between paper and iPad versions ($r(43) = 0.86$, $p < 0.001$) and no difference between TorCA Sum Index on paper ($M = 299.9$, SD = 18.1) and iPad ($M = 300.7$, SD = 18.4) versions ($t(44) = -0.56$, $p = 0.58$). There was a trend ($t(44) = 2.00$, $p = 0.052$) for the mean Sum Index to be slightly lower on the first administration ($M = 298.9$, SD = 18.6) compared to the second ($M = 301.7$, SD = 17.9). Test–retest reliability between first and second administration was good ($r(43) = 0.87$, $p < 0.001$). There was no association between test–retest interval and

Table 10 Group differences on selected Toronto Cognitive Assessment subtests

Subtest	aMCI (SD)	NC (SD)	*p* value*	Effect size, Hedge's *g* (95% CI)
Word List Learning Trials Total	14.2 (3.3)	20.8 (4.4)	0.001*	– 1.66<br>(– 2.10, – 1.22}
Word List Delayed Recall Total	1.6 (1.5)	6.0 (2.5)	0.001*	– 2.07<br>(– 2.54, – 1.60)
Word List Delayed Recognition Total	16.7 (2.2)	19.3 (1.3)	0.001*	– 1.40<br>(– 1.82, – 0.98)
Benson Figure Delayed Recall Total	5.0 (3.9)	11.0 (3.2)	0.001*	– 1.71<br>(– 2.15, – 1.26)
Clock Drawing Total	14.3 (1.8)	14.1 (1.3)	0.53	0.12<br>(– 0.26, 0.50)
Benson Figure Copy Total	15.8 (1.1)	15.7 (1.2)	0.85	0.03<br>(– 0.34, 0.41)
Digit Span Total	11.6 (2.1)	12.0 (2.0)	0.39	– 0.16<br>(– 0.54, 0.22)
Serial Subtractions Total	24.1 (2.5)	24.4 (3.0)	0.59	– 0.10<br>(– 0.48, 0.28)
Trail Making A Time to Completion (sec)	56.1 (15.5)	47.2 (19.4)	0.01	0.51<br>(0.12, 0.89)
Trail Making B Time to Completion (sec)	135.4 (54.0)	102.4 (55.6)	0.002*	0.60<br>(0.21, 0.99)
Verbal Fluency—F words Total	14.6 (4.5)	14.7 (3.8)	0.99	0.00<br>(– 0.38, 0.38)
Verbal Fluency—Animals Total	15.0 (4.3)	18.6 (5.1)	0.001*	– 0.76<br>(– 1.15, – 0.37)
MINT Naming Total	13.8 (1.3)	13.9 (2.2)	0.71	– 0.07<br>(– 0.45, 0.31)

aMCI amnestic mild cognitive impairment, *CI* confidence interval, *NC* normal cognition, *SD* standard deviation
*Significance tests corrected for multiple comparisons using Bonferroni correction at $p \leq 0.05/13$ (0.0038)

change in Sum Index on first and second testing ($r(43) = 0.04$, $p = 0.77$). In addition to lack of a linear relationship between the change in Sum Index and test–retest interval, neither a quadratic ($p = 0.90$) nor a logarithmic model ($p = 0.66$) fit the data. In addition, the mean TorCA Sum Index did not differ for the group that took the paper version first ($M = 294.7$, SD = 16.8) compared to the group that took the iPad version first ($M = 303.0$, SD = 19.6) ($t(43) = 1.5$, $p = 0.14$).

Discussion

The TorCA was administered to 303 healthy volunteers between ages 50 and 89 years, yielding a relatively brief assessment of multiple cognitive domains with median administration time of 34 min. Test–retest results remained relatively stable over a median of 73 days (range 28–120) with mean increase of only 3.3 points. Age and education accounted for only 5% of the variance in total score. Although age-adjusted norms are available for each decade from 50 to 89 years, the TorCA can be administered across this range with minimal need for age correction. Paper and iPad version scores were not significantly different. The iPad version provides easier administration with near automation of scoring and graphical representation of percentile scores (Fig. 4).

Overall stability was good with only modest increase in the Sum Index on retesting. Stability coefficients were low for Orientation, Delayed Recognition, Visuospatial Function, and Working Memory/Attention/Executive Control due to the restricted range of scores. Nevertheless, these scores demonstrated a very small percentage change in scores. The change in the Sum Index (1.1%) reflected increases in the immediate and delayed memory indices (14.3% and 10.7% respectively) with no other index exceeding an increase of 1.5% (Language).

Internal consistency of the Sum Index was adequate and reflected the heterogeneous nature of individual tests. Low internal consistency reflected the diverse nature of cognitive abilities on Delayed Recall and Working Memory/Attention/Executive Control. The former combines verbal and visual memory, whereas the latter combines heterogeneous measures related to frontal system function. Low internal consistency also reflected restricted range in scores on Orientation, Delayed Recognition, and Visuospatial Function.

Table 11 Results of backward stepwise logistic regression of Toronto Cognitive Assessment indices

	Variable in equation							95% CI for Exp(*B*)	
		B	SE	Wald	*df*	*p* value	Exp(*B*)	Lower	Upper
Step 1[a]	ORIENT	− 0.167	0.291	0.330	1	0.566	0.846	0.479	1.496
	MIR	− 0.378	0.145	6.802	1	0.009	0.685	0.516	0.910
	MDR	− 0.452	0.148	9.374	1	0.002	0.636	0.476	0.850
	MDRec	− 0.395	0.278	2.016	1	0.156	0.674	0.390	1.162
	VisSpat	0.677	0.313	4.680	1	0.031	1.969	1.066	3.637
	ExecCon	0.174	0.064	7.384	1	0.007	1.190	1.050	1.350
	Lang	0.019	0.078	0.058	1	0.810	1.019	0.875	1.186
	Constant	− 19.420	13.579	2.045	1	0.153	0.000		
Step 2	ORIENT	− 0.167	0.287	0.337	1	0.561	0.846	0.482	1.486
	MIR	− 0.370	0.140	6.936	1	0.008	0.691	0.525	0.910
	MDR	− 0.449	0.146	9.514	1	0.002	0.638	0.479	0.849
	MDRec	− 0.389	0.276	1.979	1	0.160	0.678	0.394	1.165
	VisSpat	0.672	0.309	4.719	1	0.030	1.958	1.068	3.590
	ExecCon	0.179	0.061	8.739	1	0.003	1.196	1.062	1.347
	Constant	− 18.585	12.887	2.080	1	0.149	0.000		
Step 3	MIR	− 0.374	0.139	7.283	1	0.007	0.688	0.524	0.903
	MDR	− 0.468	0.146	10.321	1	0.001	0.626	0.470	0.833
	MDRec	− 0.387	0.274	1.994	1	0.158	0.679	0.397	1.162
	VisSpat	0.677	0.309	4.810	1	0.028	1.969	1.075	3.606
	ExecCon	0.178	0.061	8.418	1	0.004	1.195	1.060	1.348
	Constant	− 20.253	12.534	2.611	1	0.106	0.000		
Step 4	MIR	− 0.407	0.132	9.486	1	0.002	0.666	0.514	0.862
	MDR	− 0.518	0.140	13.687	1	0.000	0.596	0.453	0.784
	VisSpat	0.711	0.292	5.911	1	0.015	2.035	1.148	3.609
	ExecCon	0.176	0.058	9.062	1	0.003	1.192	1.063	1.337
	Constant	− 27.256	11.044	6.091	1	0.014	0.000		

CI confidence interval, *SE* standard error
[a]Variable(s) entered in step 1: Orientation (ORIENT), Immediate Memory (MIR), Delayed Recall (MDR), Delayed Recognition (MDRec), Visuospatial (VisSpat), Working Memory/Attention/Executive Control (ExecCon), Language (Lang)

We validated the TorCA for detection of aMCI based on a need for cognitive assessment tools that can identify early decline, that are much shorter than typical neuropsychological batteries, and that can be administered by any health professional or trained assistant. A combination of TorCA subscores yielded correct classification, sensitivity, and specificity of over 90%. Logistic regression revealed that scores in four domains—Immediate Recall, Delayed Verbal and Visual Recall, Visuospatial Function, and Working Memory/Attention/Executive Control—correctly classified 92% of participants, and yielded an easily applied formula to calculate the probability of aMCI (www.tdra.ca). This is automatically calculated with the iPad version of the TorCA. It should be emphasized that the correct classification of 92% arises from four domains of the TorCA rather than the total score on the entire test. In contrast, correct classification was 79% based on the Sum Index (total score).

Although the logistic regression probability of 0.55 for aMCI is the optimal cutoff value, this may not always represent the best decision value for determining positive or negative cases. If sensitivity and specificity are held constant, PPV decreases as pretest disease probability (prevalence) decreases and increases as pretest probability increases. Conversely, NPV increases with decrease in pretest probability and decreases as pretest probability increases. PPVs and NPVs listed earlier for the optimal value relate only to the pretest probability of aMCI in our sample (50/107 = 0.47). Table 13 presents the range of PPV and NPV values for a cutoff value of 0.55 for pretest probabilities ranging from 0.05 to 0.90.

Table 12 Toronto Cognitive Assessment and neuropsychological test domain intercorrelations (Pearson *r*)

	TorCA domain					
Neuropsychological test domain	Memory immediate recall	Memory delayed recall	Memory delayed recognition	Visuospatial	Executive control[a]	Language
Immediate Recall	0.64** L	0.75** L	0.58** L	0.04	0.24* S	0.29** S
Delayed Recall	0.63** L	0.76** L	0.67** L	− 0.01	0.16	0.29** S
Delayed Recognition	0.64** L	0.75** L	0.68** L	− 0.01	0.15	0.25* S
Visuospatial	0.14	0.21* S	0.11	0.25* S	0.14	0.07
Cognitive Flexibility	0.36** M	0.34** M	0.34** M	0.13	0.33** M	0.29** S
Attention/Concentration	0.24* S	0.30** M	0.28** S	0.09	0.40** M	0.33** M
Executive Control[a]	0.48** M	0.36** M	0.26** S	− 0.06	0.52** L	0.48** M
Verbal Fluency	0.51** L	0.36** M	0.33** M	− 0.06	0.62** L	0.65** L
Language	0.24* S	0.17	0.24* S	0.03	0.52** L	0.62** L

Effect sizes: L = large, $r \geq 0.5$; M = medium, 0.30–0.49; S = small, 0.10–0.29
*$p < 0.05$; ** $p < 0.01$
[a]Working Memory/Attention/Executive Control

PPVs and NPVs for a cutoff value of 0.90 are also provided. If a logistic regression value of 0.55 or higher is obtained for individuals with pretest probability of 0.20, then 72% will be correctly classified as aMCI. However, 28% will be misclassified, which is unacceptable. At the same level of pretest probability, a logistic regression value less than 0.55 results in correctly ruling out aMCI in 98% of negative cases. At a pretest probability of 0.20, raising the "rule-in" predicted value to 0.90 results in 88% of positive cases being true aMCI with only 12% false positives. A level of 0.20 was chosen in these examples because this is approximately the estimated prevalence of aMCI in community samples [24].

Based on the validation data for TorCA Sum Index reported in this article, the TorCA is comparable to published data on the MoCA for detection of MCI. A meta-analysis of 20 studies conducted by Ciesielska et al. [25] reported that a MoCA cutoff value of 25/30 correctly yielded a sensitivity of 80% and specificity of 81%. A meta-analysis of nine studies [26] evaluating the MoCA's ability to discriminate aMCI from normal controls found that a cutoff value of 23/30 yielded a correct classification of 86% (95% CI 83–90%) with a sensitivity of 83% (95% CI 76–89%) and specificity of 88% (95% CI 84–92%), while the original cutoff value of 26/30, as suggested by Nasreddine et al. [2], yielded correct classification of only 78% (95% CI 75–82%) with sensitivity of 94% (95% CI 91–97%) and specificity of 66% (95% CI 60–71%). This compares to correct classification of 79% for the TorCA with a sensitivity and specificity of 80% and 79% using the Sum Index. The TorCA is also comparable to the Addenbrooke's Cognitive Examination (ACE-R and ACE III) based on published data [27, 28]. Ahmed et al. [27] reported that the ACE-R correctly classified 74% (95% CI 56–87%) of MCI and normal controls with a sensitivity of 90% (95% CI 58–98%) and specificity of 67% (95% CI 41–84%). Matias-Guiu et al. [28] reported that the ACE-III correctly classified 75% (95% CI 66–82%) of MCI and normal controls with a sensitivity of 77% (95% CI 62–87%) and specificity of 75% (95% CI 62–83%). Although confidence intervals were not provided in the reports by Ahmed et al. and Matias-Guiu et al. [27, 28], we calculated them for comparison to our data.

The TorCA has potential resource allocation implications in centers with neuropsychology resources by identifying patients who do not require neuropsychological assessment due to a high probability of aMCI or because this disorder is effectively ruled out. Although the logistic regression was exploratory, a reasonable strategy might be to rule out aMCI if probability, based on the logistic regression formula, is below 0.55. Due to the likelihood that the logistic regression formula overestimates classification [29], we recommend a value of 0.90 or higher to rule in aMCI. For values between 0.55 and 0.90, referral should preferably be made for neuropsychological assessment to confirm diagnosis. In the absence of available neuropsychology resources, these patients should be followed to establish diagnosis.

The probability of aMCI is: 93.7%

NB: Probability of aMCI applies only to differentiation of aMCI vs normal cognition

Total Score:
295

Index Summary:

Results Summary

Age	SUM	ORIENT	MIR	MDR	MDRec	VisSpat	ExecCon	Lang
75	295	12	18	11	18	31	119	86

Percentile Rating

Age Group	SUM	ORIENT	MIR	MDR	MDRec	VisSpat	ExecCon	Lang
50-89	50 ★	50 ★	16 ●	9 ●	5 ▼	84 ★	91 ★	63 ★
70-79	50 ★	50 ★	25 ★	9 ●	5 ▼	91 ★	95 ★	63 ★

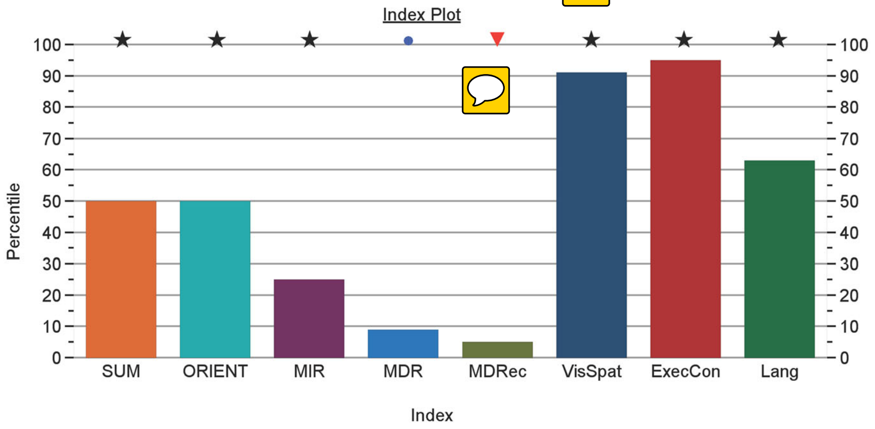

★ Within normal limits
● Borderline
▼ Below normal limits

SUM	Total Score
ORIENT	Orientation
MIR	Immediate memory
MDR	Delayed recall
MDRec	Delayed recognition
VisSpat	Visuospatial
ExecCon	Executive control
Lang	Language

Fig. 4 iPad summary score sheet showing domain scores and numerical and graphic percentile ratings. Probability of aMCI shown as 93.7%. aMCI amnestic mild cognitive impairment

Study limitations should be acknowledged. First is the need for cross-validation. Whereas the validation study revealed that the use of the logistic regression formula would refine the identification of aMCI, this represents an initial, exploratory result and further cross-validation of the formula is needed to confirm critical values and stability of constituent indices. A second limitation is that the logistic regression formula for probability of aMCI applies only to differential diagnosis of aMCI vs normal aging. Future studies are needed to validate the TorCA for differentiating aMCI from other cognitive disorders, and to determine whether it performs equally well for identifying single vs multiple domain aMCI. A third limitation is that participants in the validation study had relatively high IQs. Studies are needed to determine validity of the TorCA for diagnosing aMCI in participants with lower IQs. In addition, a caution is that interpretation of positive or negative cases must take into account differences between patients' estimated pretest probabilities of a condition and prevalence of the condition in validation studies. A fourth limitation is that the orientation items consisting of Prime Minister, Premier, and season are country specific. This will be addressed in future by translating the TorCA into languages other than English and carrying out normative and validation studies using the translated tests. Ideally, normative and validation studies should also be carried out in English-speaking countries other than Canada. Finally, this study focused only on aMCI from a diagnostic perspective. Future studies will be needed to validate the TorCA for diagnosis of other forms of mild cognitive decline. It is likely that the discriminating indices on the TorCA will differ from those that predict aMCI.

Table 13 Positive and negative predictive values

	Probability obtained from logistic regression formula			
	p(aMCI) = 0.55		*p*(aMCI) = 0.90	
Pretest probability	PPV	NPV	PPV	NPV
0.05	0.35	0.995	0.60	0.98
0.10	0.53	0.99	0.76	0.96
0.20	0.72	0.98	0.88	0.90
0.30	0.81	0.96	0.93	0.84
0.40	0.87	0.94	0.95	0.78
0.50	0.91	0.92	0.97	0.70
0.60	0.94	0.88	0.98	0.61
0.70	0.96	0.83	0.98	0.61
0.80	0.98	0.74	0.99	0.37
0.90	0.99	0.56	0.996	0.21

PPV and NPV shown for varying pretest probabilities and two probability values for detecting aMCI, 0.55 and 0.90, obtained by logistic regression described in text
aMCI amnestic mild cognitive impairment, *PPV* positive predictive value, *NPV* negative predictive value

Conclusions

The TorCA is a relatively short cognitive assessment tool for identification of early cognitive decline and can be administered by any health care professional or assistant with appropriate training. It also has the potential to save both time and physical resources by identifying patients who may not require neuropsychological assessments for diagnosing aMCI. Future studies will focus on cross-validating the TorCA for aMCI and validating this test for disorders other than aMCI.

Abbreviations

aMCI: Amnestic mild cognitive impairment; KBNA: Kaplan–Baycrest Neurocognitive Assessment; LR: Logistic regression; LRNR: Likelihood ratio of a negative response; LRPR: Likelihood ratio of a positive response; MINT: Multilingual Naming Test; MoCA: Montreal Cognitive Assessment; NC: Normal cognition; NPV: Negative predictive value; PPV: Positive predictive value; RRI: Rotman Research Institute; TorCA: Toronto Cognitive Assessment; WAIS-III: Wechsler Adult Intelligence Scale—III; WASI: Wechsler Abbreviated Scale of Intelligence; WMS-R: Wechsler Memory Scale—Revised

Acknowledgements

The authors would like to acknowledge S. Wilson for comprehension and semantic knowledge tasks, T. Gollan for the abbreviated naming task (MINT), A. Hillis for the repetition task, K. Possin and J. Kramer for the Benson Figure, the Frontotemporal Dementia (FTLD) Workgroup (Chair, D. Knopman) of the National Alzheimer's Coordinating Center (NACC) (Director, W.A. Kukull, Grant Number U01 AG016976) for use of the repetition task and the Benson Figure from the NACC FTLD Module, and G.G. Fillenbaum for CERAD use and materials.

Funding

This work was supported in part by the Toronto Dementia Research Alliance Partner Institutions (Baycrest Health Sciences, Centre for Addiction and Mental Health, St. Michael's Hospital, Sunnybrook Health Sciences Centre, University Health Network, and Faculty of Medicine, University of Toronto); Department of Medicine Alternative Funding Plan and Division of Neurology Innovation Fund, University of Toronto; Edwards Family Foundation; and Ontario Neurodegenerative Disease Research Initiative (ONDRI) funded by Ontario Brain Institute (OBI). MF receives support from the Saul A. Silverman Family Foundation as a Canada International Scientific Exchange Program and Morris Kerzner Memorial Fund. SEB receives support from the Brill Chair in Neurology, University of Toronto and Sunnybrook Foundation, the Hurvitz Brain Sciences Research Program, Sunnybrook Research Institute, and the Department of Medicine, Sunnybrook Health Sciences Centre. SD holds the Dalhousie Medical Research Foundation Irene MacDonald Sobey Endowed Chair in Curative Approaches to Alzheimer's Disease. GN receives support from the George, Margaret and Gary Hunt Family Chair in Geriatric Medicine, University of Toronto. SCS, RS, and TG received partial grant support from CIHR MOP 201403, the Ontario Brain Institute, and Brain Canada. The funding sources had no role in the study design, in the collection, analysis and interpretation of data, in writing of the report, and in the decision to submit data for publication.

Availability of data and materials

The datasets used and/or analyzed during the current study are available from the corresponding author on reasonable request.

Authors' contributions

MF, LL, SEB, MCT, and DFT-W contributed to the conception of the TorCA. MF, LL, SEB, KAS, and DFT-W contributed to the design of the normative and validation studies, and interpretation of the data. LL carried out the data analyses. MF, LL, and DFT-W prepared the initial draft of the manuscript with contributions from KAS and YG related to the validation study. MCT designed the language, visual, and verbal memory portions of the TorCA and contacted S. Wilson, T. Gollan, A. Hillis, K. Possin, J. Kramer, the Frontotemporal Dementia (FTLD) Workgroup of the National Alzheimer's Coordinating Center (NACC), and G.G. Fillenbaum for use of specific tasks incorporated into the TorCA as listed in the Acknowledgements. MF, LL, MCT, KAS, YG, RS, NN, TG, SCS, MOA, MB, SD, AF, CEF, JF, BDG, MG, NH, RK, JK, SK, BL, SL, MPM, GN, RP, TKR, WR, MUW, NPLGV, JLW, SEB, and DFT-W reviewed and approved the final manuscript. DFT-W developed the initial iPad version of the TorCA. RS, NN, TG, SCS, and RP contributed to the final development of the iPad version. LL, MF, KAS, YG, MOA, AF, MG, JK, and JLW contributed to data collection.

Authors' information

Tom Gee is now at Indoc Research, Toronto, ON, Canada. Barry D. Greenberg is now at Department of Neurology, Johns Hopkins University School of Medicine, Baltimore, MD, USA.

Ethics approval and consent to participate

The study was approved by the Research Ethics Board at Baycrest Health Sciences. Written informed consent was obtained from all participants.

Competing interests

MF received financial support for a Behavioural Neurology fellow from Eli Lilly Canada, served on an advisory board for Eli Lilly Canada, receives royalties for a book on Clock Drawing from Oxford University Press, is listed on a provisional patent related to methods and kits for differential diagnosis of Alzheimer's disease vs frontotemporal dementia using blood biomarkers, and may be listed on the planned patent application, and serves on the editorial board of *Brain and Cognition*. LL receives royalties from Pearson Assessment on sales of the Kaplan Baycrest Neurocognitive Assessment (KBNA). NH received research support from Axovant, Lundbeck and Roche, and consultation fees from Merck, Lilly, Mediti and Astellas. SEB reports institutional grants from Pfizer, GE Healthcare, Eli Lilly, Roche, Cognoptix, Biogen, and Novartis and personal honoraria from Pfizer, Eli Lilly, Boehringer Ingelheim, Novartis, Merck, and Medscape (Biogen Idec); SEB also reports salary support from Sunnybrook Research Institute, Brill Chair, Department of Medicine, Sunnybrook Health Sciences Centre. SCS is Chief Science Officer of ADMdx, LLC. MCT, KAS, YG, RS, NN, TG, MOA, MB, SD, AF, CEF, JF, BDG, MG, RK, JK, SK, BL, SL, MPM, GN, RP, TKR, WR, MUW, NPLGV, JLW, and DFT-W do not have any competing interests.

Publisher's Note

Springer Nature remains neutral with regard to jurisdictional claims in published maps and institutional affiliations.

Author details

[1]Department of Medicine (Neurology), University of Toronto, Toronto, ON, Canada. [2]Baycrest Health Sciences, 3560 Bathurst Street, Toronto, ON M6A 2E1, Canada. [3]Rotman Research Institute of Baycrest Centre, Toronto, ON, Canada. [4]Toronto Dementia Research Alliance, Toronto, ON, Canada. [5]Mt. Sinai Hospital, Toronto, ON, Canada. [6]Department of Psychology, Glendon

College, Toronto, ON, Canada. [7]Toronto Western Hospital, University Health Network, Toronto, ON, Canada. [8]Tanz Centre for Research in Neurodegenerative Diseases, Toronto, ON, Canada. [9]Sunnybrook Research Institute, Toronto, ON, Canada. [10]Department of Medical Biophysics, University of Toronto, Toronto, ON, Canada. [11]Canada International Scientific Exchange Program, Toronto, ON, Canada. [12]Lawson Health Research Institute, London, ON, Canada. [13]Parkwood Institute, London, ON, Canada. [14]Department of Medicine (Neurology and Geriatric Medicine) and Department of Medical Neuroscience, Dalhousie University, Halifax, NS, Canada. [15]Keenan Research Centre for Biomedical Research, Li Ka Shing Knowledge Institute, St. Michael's Hospital, Toronto, ON, Canada. [16]University Health Network, Toronto, ON, Canada. [17]Sunnybrook Health Sciences Centre, Toronto, ON, Canada. [18]Department of Psychiatry, University of Toronto, Toronto, ON, Canada. [19]Centre for Addiction and Mental Health, Toronto, ON, Canada. [20]Hurvitz Brain Sciences Program, Sunnybrook Health Sciences Centre, Toronto, ON, Canada. [21]Krembil Research Institute, University Health Network, Toronto, ON, Canada. [22]Department of Psychology, University of Toronto, Toronto, ON, Canada. [23]Department of Medicine (Geriatric Medicine) and Institute of Health Policy, University of Toronto, Toronto, ON, Canada. [24]Campbell Family Mental Health Research Institute, Toronto, ON, Canada. [25]LC Campbell Cognitive Neurology Research Unit, Toronto, ON, Canada.

Received: 25 January 2018 Accepted: 8 May 2018
Published online: 18 July 2018

References

1. Folstein MF, Folstein SE, McHugh PR. 'Mini-mental State'. A practical method for grading the cognitive state of patients for the clinician. J Psychiatr Res. 1975;12:189–98. https://doi.org/10.1016/0022-3956(75)90026-6.
2. Nasreddine ZS, Phillips NA, Bédirian V, Charbonneau S, Whitehead V, Collin I, et al. The Montreal Cognitive Assessment, MoCA: a brief screening tool for mild cognitive impairment. J Am Geriatr Soc. 2005;53:695–9. https://doi.org/10.1111/j.1532-5415.2005.53221.x.
3. Darvesh S, Leach L, Black SE, Kaplan E, Freedman M. The Behavioural Neurology Assessment. Can J Neurol Sci. 2005;32:167–77. https://doi.org/10.1017/S0317167100003930.
4. Morris JC, Mohs RC, Rogers H, Fillenbaum G, Heyman A. Consortium to Establish a Registry for Alzheimer's Disease (CERAD) clinical and neuropsychological assessment of Alzheimer's disease. Psychopharmacol Bull. 1988;24:641–52.
5. Possin KL, Laluz VR, Alcantar OZ, Miller BL, Kramer JH. Distinct neuroanatomical substrates and cognitive mechanisms of figure copy performance in Alzheimer's disease and behavioral variant frontotemporal dementia. Neuropsychologia. 2011;49:43–8. https://doi.org/10.1016/j.neuropsychologia.2010.10.026.
6. Freedman M, Leach L, Kaplan E, Winocur G, Shulman KI, Delis DC. Clock Drawing: A Neuropsychological Analysis. New York, New York: Oxford University Press; 1994.
7. Henri-Bhargava A, Stuss DT, Freedman M. Function and dysfunction of the prefrontal lobes in neurodegenerative diseases. In: Gediminas PE, editor. Progressive Cognitive Impairment and its Neuropathologic Correlates. New York: Nova Science Publishers Inc; 2016. p. 51–68.
8. Army Individual Test Battery: Manual of Directions and Scoring. Washington, DC: War Department, Adjutant General's Office; 1944.
9. Gollan TH, Weissberger GH, Runnqvist E, Montoya RI, Cera CM. Self-ratings of spoken language dominance: a multilingual naming test (MINT) and preliminary norms for young and aging Spanish-English bilinguals. Biling Lang Cogn. 2012;15:594–615. https://doi.org/10.1017/S1366728911000332.
10. Leach L, Kaplan E, Rewilak D, Richards B, Proulx GB. Kaplan Baycrest Neurocognitive Assessment Manual. San Antonio, TX: The Psychological Corporation; 2000.
11. Wechsler D. Wechsler Adult Intelligence Scale—Third Edition. San Antonio, TX: The Psychological Corporation; 1997.
12. Wechsler D. Wechsler Memory Scale—Revised. San Antonio, TX: The Psychological Corporation; 1987.
13. Wechsler D. Wechsler Abbreviated Scale of Intelligence. San Antonio, TX: The Psychological Corporation; 1999.
14. Kaplan E, Goodglass H, Weintraub S. The Boston Naming Test. Philadelphia, PA: Lea & Febiger; 1983.
15. Delis DC, Kaplan E, Kramer JH. The Delis-Kaplan Executive Function System (D-KEFS). San Antonio, TX: The Psychological Corporation; 2001.
16. Troyer AK, Rich JB. Psychometric properties of a new metamemory questionnaire for older adults. J Gerontol B Psychol. 2002;57:19–27. https://doi.org/10.1093/geronb/57.1.P19.
17. Lawton MP, Brody EM. Assessment of older people: self-maintaining and instrumental activities of daily living. Gerontologist. 1969;9:179–86. https://doi.org/10.1093/geront/9.3_Part_1.179.
18. Snaith RP. The Hospital Anxiety and Depression Scale. Health Qual Life Out. 2003;1:29. https://doi.org/10.1186/1477-7525-1-29.
19. Albert MS, DeKosky ST, Dickson D, Dubois B, Feldman HH, Fox NC, et al. The diagnosis of mild cognitive impairment due to Alzheimer's disease: recommendations from the National Institute on Aging-Alzheimer's Association workgroups on diagnostic guidelines for Alzheimer's disease. Alzheimers Dement. 2011;7:270–9. https://doi.org/10.1016/j.jalz.2011.03.008.
20. Cohen J. Statistical power analysis for the behavioral sciences. 2nd ed. Hillsdale, NJ: Lawrence Erlbaum Associates; 1988.
21. McHugh ML. Interrater reliability: the kappa statistic. Biochem Medica. 2012;22:276–82. http://dx.doi.org/10.11613/BM.2012.031.
22. Jaeschke R, Guyatt GH, Sackett DL. Users' guides to the medical literature. III. How to use an article about a diagnostic test. B. What are the results and will they help me in caring for my patients? The Evidence-Based Medicine Working Group. JAMA. 1994;271:703–7.
23. Hawkins RC. The Evidence Based Medicine approach to diagnostic testing: practicalities and limitations. Clin Biochem Rev. 2005;26:7–18.
24. Petersen RC, Roberts RO, Knopman DS, Boeve BF, Geda YE, Ivnik RJ, et al. Mild cognitive impairment: ten years later. Arch Neurol. 2009;66:1447–55. https://doi.org/10.1001/archneurol.2009.266.
25. Ciesielska N, Sokolowski R, Mazur E, Podhorecka M, Polak-Szabela A, Kedziora-Kornatowska K. Is the Montreal Cognitive Assessment (MoCA) test better suited than the Mini-Mental State Examination (MMSE) in mild cognitive impairment (MCI) detection among people aged over 60? Meta-analysis. Psychiatr Pol. 2016;50:1039–52. https://dx.doi.org/10.12740/PP/45368.
26. Carson N, Leach L, Murphy KJ. A re-examination of Montreal Cognitive Assessment (MoCA) cutoff scores. Int J Geriatr Psychiatry. 2018;33:379–88. https://doi.org/10.1002/gps.4756.
27. Ahmed S, de Jager C, Wilcock G. A comparison of screening tools for the assessment of mild cognitive impairment: preliminary findings. Neurocase. 2012;18:336–51. https://dx.doi.org/10.1080/13554794.2011.608365.
28. Matias-Guiu JA, Cortes-Martinez A, Valles-Salgado M, Rognoni T, Fernandez-Matarrubia M, Moreno-Ramos T, et al. Addenbrooke's cognitive examination III: diagnostic utility for mild cognitive impairment and dementia and correlation with standardized neuropsychological tests. Int Psychogeriatr. 2017;29:105–13. https://dx.doi.org/10.1017/S1041610216001496.
29. Steyerberg EW, Eijkemans MJ, Habbema JD. Stepwise selection in small data sets: a simulation study of bias in logistic regression analysis. J Clin Epidemiol. 1999;52:935-42. https://doi.org/10.1016/S0895-4356(99)00103-1.

Ready to submit your research? Choose BMC and benefit from:

- fast, convenient online submission
- thorough peer review by experienced researchers in your field
- rapid publication on acceptance
- support for research data, including large and complex data types
- gold Open Access which fosters wider collaboration and increased citations
- maximum visibility for your research: over 100M website views per year

At BMC, research is always in progress.

Learn more biomedcentral.com/submissions

BMC
